# Reporting Items for Updated Clinical Guidelines: Checklist for the Reporting of Updated Guidelines (CheckUp)

**DOI:** 10.1371/journal.pmed.1002207

**Published:** 2017-01-10

**Authors:** Robin W. M. Vernooij, Pablo Alonso-Coello, Melissa Brouwers, Laura Martínez García, CheckUp Panel

**Affiliations:** 1 Iberoamerican Cochrane Centre, Biomedical Research Institute Sant Pau (IIB Sant Pau), Barcelona, Spain; 2 CIBER of Epidemiology and Public Health (CIBERESP), Spain; 3 Clinical Epidemiology and Biostatistics Department, McMaster University, Hamilton, Canada; 4 Program in Evidence-based Care, Cancer Care Ontario, Hamilton, Canada

## Abstract

**Background:**

Scientific knowledge is in constant development. Consequently, regular review to assure the trustworthiness of clinical guidelines is required. However, there is still a lack of preferred reporting items of the updating process in updated clinical guidelines. The present article describes the development process of the Checklist for the Reporting of Updated Guidelines (CheckUp).

**Methods and Findings:**

We developed an initial list of items based on an overview of research evidence on clinical guideline updating, the Appraisal of Guidelines for Research and Evaluation (AGREE) II Instrument, and the advice of the CheckUp panel (*n* = 33 professionals). A multistep process was used to refine this list, including an assessment of ten existing updated clinical guidelines, interviews with key informants (response rate: 54.2%; 13/24), a three-round Delphi consensus survey with the CheckUp panel (33 participants), and an external review with clinical guideline methodologists (response rate: 90%; 53/59) and users (response rate: 55.6%; 10/18). CheckUp includes 16 items that address (1) the presentation of an updated guideline, (2) editorial independence, and (3) the methodology of the updating process. In this article, we present the methodology to develop CheckUp and include as a supplementary file an explanation and elaboration document.

**Conclusions:**

CheckUp can be used to evaluate the completeness of reporting in updated guidelines and as a tool to inform guideline developers about reporting requirements. Editors may request its completion from guideline authors when submitting updated guidelines for publication. Adherence to CheckUp will likely enhance the comprehensiveness and transparency of clinical guideline updating for the benefit of patients and the public, health care professionals, and other relevant stakeholders.

## Abstract 2

Pablo Alonso-Coello and colleagues describe a checklist to guide the reporting of clinical guideline updates.

## Background

Trustworthy clinical guidelines aim to assist decision making by providing recommendations that are informed by the best available evidence and include an assessment of the benefits and harms of alternative care options [1,2]. Because of the continuous emergence of new research evidence (i.e., changes in available interventions, effects, or cost) [3], appropriate updating to maintain the trustworthiness of clinical guidelines is challenging since it requires regular surveillance and reviewing of the new evidence [4,5].

Updating clinical guidelines is a process that includes different stages: (1) prioritisation of candidate guidelines or recommendations to update [6], (2) identification of new scientific evidence [3,6–8], (3) assessment of the need to update [3,6,9], (4) updating the recommendations [6,10–12], and (5) publication of the updated guideline [6,13]. However, there is no consensus about what is the optimal methodology to operationalise each of these steps or how to report on the process [5,14,15,16]; the available guidance from guideline institutions is suboptimal [17,18].

Trustworthiness standards for guidelines have been published by both the Institute of Medicine (IOM) and the Guidelines International Network (G-I-N) [1,2]. Additionally, instruments are available for assessing the quality of clinical guidelines, such as the Appraisal of Guidelines for Research and Evaluation (AGREE) II Instrument [19], while others, such as the GIN-McMaster Guideline Development Checklist [20], support developing and implementing trustworthy clinical guidelines. However, guideline updating requires some different methodological considerations and unique communication procedures. Currently, none of the existing tools address these issues.

To address this gap, in a partnership of the Iberoamerican Cochrane Centre (www.cochrane.org), the AGREE Collaboration (www.agreetrust.org), and the G-I-N Updating Guidelines Working Group (www.g-i-n.net/working-groups/updating-guidelines), we have developed the Checklist for the Reporting of Updated Guidelines (CheckUp). This article about CheckUp is targeted at guideline developers and users of guidelines. In the article, we present the methodology of the development process and the final checklist. In a supplementary file, we present explanations and examples for each item (S1 Appendix).

## Methodology

**Fig 1:**
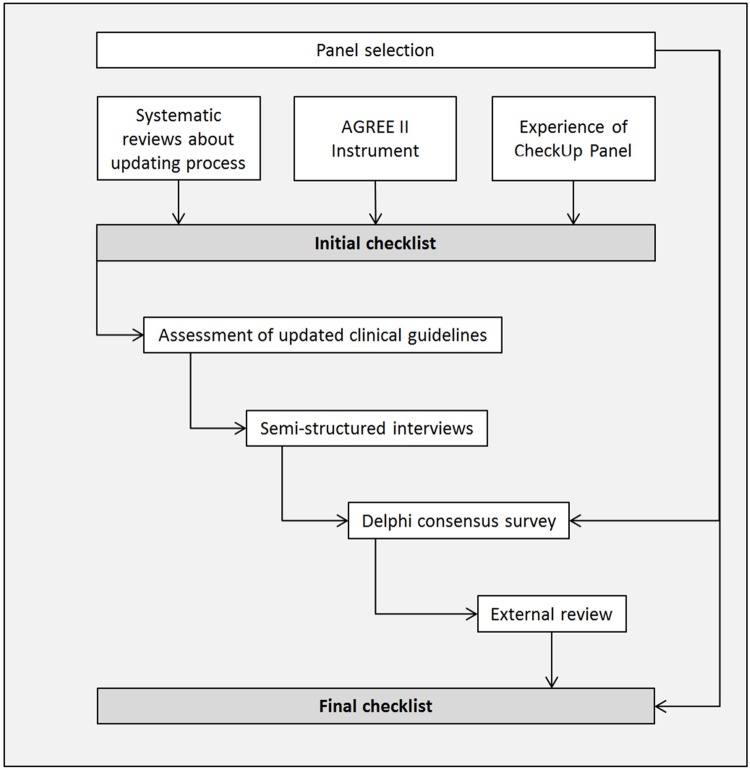
Checklist development process. Abbreviation: AGREE, Appraisal of Guidelines for Research and Evaluation.

For reporting the development process of CheckUp, we followed Enhancing the QUAlity and Transparency Of health Research(EQUATOR) and Moher’s criteria [21,22]. The development of CheckUp consisted of four phases: (1) panel selection, (2) generation of the initial checklist, (3) optimisation of the checklist, and (4) approval of the final checklist (Fig 1).

### Panel Selection

To advise on the development of the CheckUp, a panel comprising individuals with relevant experience in clinical guideline development and updating and/or in systematic reviews/guidelines research methodology was convened. Invited panel participants were identified based on a review of the main authors in the field, as well as the AGREE Trust (www.agreetrust.org) and the G-I-N (www.g-i-n.net) members. The purpose of the panel was to provide expert advice during the development process and to participate in the Delphi survey. A core group (RWMV, PAC, MB, and LMG) was established to design the protocol and provide more time-sensitive and operational advice.

### Generation of the Initial Checklist

The core group first developed an initial list of items—including explanation and examples—through brainstorming and discussion, taking into account (1) research evidence in the field [15,16,18], (2) the AGREE II Instrument [19], and (3) the panel experience. We used three core updating publications as a starting point from which the initial version of the checklist was generated [15,16,18]. These studies include an overview of the available guidance from guideline methodological handbooks [18], a systematic review of the published methodological research [16], and an international survey about the experiences of the main guideline developers [15].

### Optimisation of the Checklist

We optimised the initial checklist through a multistep process that included an assessment of existing updated clinical guidelines, semistructured interviews, a Delphi consensus survey, and an external review with clinical guideline methodologists and users (Fig 1).

#### Assessment of updated clinical guidelines

We piloted the initial checklist among a convenience sample of updated clinical guidelines to assess the used terminology, to identify missing items, and as a first step to explore its face validity. We included updated clinical guidelines that were (1) developed by G-I-N members, (2) published in English or Spanish, and (3) published between 2011 and 2013. We searched the G-I-N library (www.g-i-n.net) and the National Guideline Clearinghouse (www.guidelines.gov). Two reviewers (RWMV and SPS) applied the checklist, solving disagreements by consensus. The core group discussed the results and refined the initial list of items.

#### Semistructured interviews

To refine the checklist and to identify missing items, we conducted semistructured interviews with clinical guideline experts. We chose a convenience sample of participants, outside the CheckUp panel, with (1) experience in updating clinical guidelines, defined as having participated in the updating process of at least one clinical guideline over the past year, and (2) fluency in English. We identified the participants by contacting professionals associated with G-I-N or researchers in the field. When someone did not respond or could not participate, a new person was recruited. We continued to recruit participants and collect data until data saturation was achieved.

In each interview, participants were asked about their experiences and challenges in updating clinical guidelines. Subsequently, the participant was prompted by the interviewer (RWMV) to reflect on the strengths, weaknesses, missing concepts, and redundancies in the checklist. The interviews were audiotaped, and key themes were identified. The core group discussed the results and refined the list of items.

#### Delphi consensus survey

We reviewed the refined list of items through a Delphi consensus survey [23], with all members of the CheckUp panel. The Delphi participants assessed the inclusion, comprehensiveness, clarity, and coverage of each item and tried to identify potentially additional items for the checklist.

Using a seven-point Likert scale (one meaning strongly disagree and seven meaning strongly agree) [24], we asked participants to rate whether the item should be included in the checklist. For each item, participants were asked whether their perceptions of (1) the completeness, (2) the usability, and (3) the quality of a clinical guideline would be influenced if the item was reported. We included a free text box for suggestions to modify the items, the explanation, or the examples. We used online software to design the survey and to collect the responses (www.surveymonkey.com).

We calculated the median score for inclusion, completeness, usability, and quality for each item and classified them into (1) items with a median score of 0 to 3 points, which were excluded; (2) items with a median score of 4 to 5 points or with substantial comments that needed important revision, which were retained, modified, and further tested; and (3) items with a median score of 6 to 7 points and without substantial comments, which were included and not evaluated further in the following rounds.

One reviewer (RWMV) analysed the quantitative and qualitative results and suggested potential solutions. The core group discussed the results and potential solutions and refined the list of items accordingly. We continued with additional rounds until consensus for inclusion or exclusion was reached and no more relevant comments were provided.

#### External review with clinical guideline methodologists

To evaluate the usability of the checklist, we conducted a survey with clinical guideline methodologists who had experience in updating clinical guidelines, as measured by having participated in the updating process of at least one clinical guideline over the past year. We also invited all of the G-I-N institutional member contacts to participate in the external review. If the contact person was not able to participate, we asked them to provide contact details of another expert working at the same institution.

Using a seven-point scale (one meaning strongly disagree and seven meaning strongly agree), we asked participants to rate the usability of each item and its influence on the confidence in an updated clinical guideline if the item was reported. A free text option was included for suggestions to modify the items, the explanation, or the examples. We used online software to design the survey and to collect the responses (www.surveymonkey.com).

We calculated the median score for usability and confidence for each item. One reviewer (RWMV) analysed the quantitative and qualitative results and suggested potential solutions. The core group discussed the results and potential solutions and refined the list of items accordingly.

#### External review with clinical guideline users

We conducted semistructured interviews with clinical guideline users to evaluate the usability of the checklist. We engaged individuals who were (1) health care professionals who used clinical guidelines in clinical practice and (2) located in Canada, Spain, or the Netherlands. We identified the participants with the help of the panel members. When someone did not respond or could not participate, a new person was recruited. We continued to recruit participants and collect data until the information was repeated and no new information emerged (data saturation).

For each interview, participants were asked whether reporting of the item in an updated clinical guideline would increase their confidence in the guideline. The participant and interviewer (RWMV) reviewed the checklist, and the participants were prompted to consider missing concepts, redundancy, and the usability of the checklist. The interviews were audiotaped, and key themes were identified. The core group discussed the results and refined the list of items.

#### Approval of the final checklist

The checklist was presented and discussed in a workshop at the 2015 G-I-N Conference in Amsterdam. In this workshop, we asked the participants whether the checklist was deemed adequate for assessment of the updated clinical guidelines [25]. We also asked the participants to give an overall impression of the checklist. The core group discussed the results and agreed on the final list of items.

## Results

### CheckUp Panel

Fifty-six potential individuals were invited to be part of the CheckUp Panel. In total, 33 professionals, 20 males and 13 females, (response rate: 58.9%, 33/56) confirmed their participation (17 from Europe, 9 from South America, 5 from North America, and 2 from Oceania). The primary role of the panellists was health care researcher (60.6%; 20/33), guideline developer (30.3%; 10/33), and clinical guideline user (9.1%; 3/33).

### Generation of the Initial Checklist

The initial checklist included 13 items within the following domains: updating rationale, scope and purpose of the updated clinical guideline, participants in the updating panel, conflicts of interest, updating methodology, differentiating original and new information, and reasons for the changes in the recommendations.

### Optimisation of the Checklist

#### Assessment of updated clinical guidelines

Initially, we assessed a convenience sample of ten updated clinical guidelines from the G-I-N library with the initial checklist [26–35]. The items more frequently reported in the included clinical guidelines were related to the literature search strategy (60%), the composition of the panel (50%), and the external review (40%). The other checklist items (e.g., assessment for the need of updating, evidence selection, rationale for updating, and rationale for changes) were reported in less than 20% of the included clinical guidelines. No additional concepts or items emerged (Table 1).

**Table 1:** CheckUp: Stages of the optimisation process (objective, sample, and results by optimisation processes).

Stage	Objectives	Sample (*n*)	Main Results
**1. Assessment of updated clinical guidelines**	Assess whether the terminology was consistent between the checklist and updated clinical guidelines Identify items that were lacking in the checklist.	Updated clinical guidelines (10).	No items were modified. No new items were added.
**2. Semistructured interviews**	Explore challenges and issues regarding the clinical guideline updating process. Identify items that were lacking in the checklist.	Clinical guideline updating process experts (13).	Five items had major modifications. Four new items were added.
**3. Delphi consensus survey**	Assess the inclusion, comprehensiveness, clarity, and coverage of each item. Identify items that were lacking in the checklist.	Clinical guideline updating process experts (33) (CheckUp panel).	All items, explanations, and examples had minor modifications regarding writing style. Two items were combined. No new items were added.
**4. External review**	Evaluate the usability of the checklist.	Clinical guideline methodologists (53).	All items, explanations, and examples had minor modifications regarding writing style.
		Clinical guideline users (10).	No items were modified.

#### Semistructured interviews

We conducted semistructured interviews with clinical guideline developers (5 from Europe, 5 from North America, and 3 from South America). In total, we interviewed 13 participants, at which point saturation was reached. As a result, we modified five items and added four new ones. The modifications were related to (a) differences in the objectives, purpose, or aim between the original and updated version; (b) the identification of new evidence; (c) the rationale for changing recommendations; and (d) the funding. The new items were related to (a) the scope of the update (partial or complete), (b) the target audience, (c) the changes in the recommendations, and (d) the plans and methodology reported to update the clinical guideline (Table 1).

#### Delphi consensus survey

All the members of the CheckUp panel (*n* = 33) were invited to participate in the Delphi consensus survey. Twenty-seven (82%) members participated in the first Delphi round, thirty-one members (93.9%) in the second Delphi round, and all (100%) members in the third and final round.

In the first round, the participants provided substantial feedback on various items, their explanation, and the accompanying examples. This feedback triggered modification in the order of the items, phrasing of the items, explanations, and examples (Table 1). All items met the inclusion criteria, and no major comments were reported. The median score for whether the participants believed that an updated clinical guideline would be more complete, usable, and of higher quality whenever the item was reported was six for all questions.

In the second round (*n* = 17 items), the amount of feedback was substantially smaller than in the first round (Table 1). We merged some items, and the checklist was reduced to 16 items. Again, all items met the inclusion criteria, and no major comments were reported. The median score for whether the participants believed the clinical guideline would be more complete, usable, and of higher quality was 7.0, 6.0, and 6.0, respectively.

In the third and last round, a general consensus was reached for all items, explanations, and examples. The median score for item inclusion, completeness, usability, and quality was ≥6 in all items (except for two items with a median score of 5.5 in the usability and quality question) (Table 2).

**Table 2:** Results of the Delphi survey (third round).

Item	Median Score for Inclusion^a^ (0–7)*	Median Score for Completeness^b^ (0–7)*	Median Score for Usability^c^ (0–7)*	Median Score for Quality^d^ (0–7)*
1. Distinguishing the updated and original version.	7	6	6	6
2. Reviewed and changed sections.	6	6	6	5
3. Presentation of new, modified, or not changed recommendations.	7	6	6	6
4. Working group of the updating process.	6	6	5	6
5. Rationale for updating the guideline.	6	6	5	6
6. Differences in the scope and purpose between the updated and the original guideline.	6	6	6	5.5
7. Reporting and justification of changes in the recommendations.	6	6.5	6	6
8. Methods for searching and identifying new evidence.	7	7	6	7
9. Methods for evidence selection.	6.5	7	6	7
10. Methods to assess the quality of the included evidence.	7	6	6	6
11. Methods for evidence synthesis.	6	6	6	6
12. Methods and plan for implementing the changes.	6	6	6	6
13. Methods for external reviewing.	6	6	5.5	6
14. Plan and methods for updating in the future.	6	6	5	6
15. Conflicts of interests of the updating group.	7	7	6	7
16. Role of the funding body.	6.5	6.5	6	6.5

^a^Should this item be included in the reporting checklist for updated clinical guidelines?;

^b^Whether this information was present or not would influence your perceptions of the completeness of the reporting of an updated clinical guideline;

^c^Whether this information was present or not would influence your perceptions of the usability of an updated clinical guideline;

^d^Whether this information was present or not would influence your perceptions of the quality of an updated clinical guideline.

* Seven-point Likert scale (0 meaning ¨strongly disagree¨ and 7 meaning ¨strongly agree¨).

#### External review

*External review with clinical guideline methodologists*. We conducted a survey with 53 clinical guideline methodologists (53/59, response rate 90%). The median scores of usability and confidence for each item were ≥6 (Table 3). Participants provided comments that improved the writing style of the items, explanations, and examples (Table 1).

**Table 3:** Results of the external review with clinical guideline methodologists.

Item	Median Score for Usability^a^ (0–7)*	Median Score for Confidence^b^ (0–7)*
1. The updated version is distinguished from the previous version of the guideline.	6	6
2. The sections reviewed in the updating process are described.	7	6.5
3. The recommendations are clearly presented and labelled as new, modified, or no change. Deleted recommendations are clearly noted.	6	6
4. The panel participants in the updated version are described.	6	6
5. The rationale for updating the guideline is reported.	6	6
6. Changes in the scope and purpose between the updated and original version are described and justified.	6.5	6
7. Changes in the original recommendations are reported and justified.	6	6
8. The methods used for searching and identifying new evidence in the updating process are described.	7	7
9. The methods used for evidence selection in the updating process are described.	7	7
10. The methods used to assess the quality of the included evidence in the updating process are described.	7	7
11. The methods used for the evidence synthesis in the updating process are described.	6	6
12. The methods and plan for implementing the changes of the updated version in practice are described.	5	5
13. The methods used for externally reviewing the updated version are described.	6	6
14. The plan and methods for updating the new version in the future are reported.	6	6
15. The conflicts of interests of the group responsible for the updated version are recorded.	7	7
16. The role of the funding body for the updated guideline is identified and described.	7	7

^a^This item is useful to evaluate an updated clinical guideline;

^b^I have more confidence in an updated clinical guideline if this item is reported.

* Seven-point scale (0 meaning ¨strongly disagree¨ and 7 meaning ¨strongly agree¨).

*External review with clinical guideline users*. We had conducted semistructured interviews with 10 clinical guideline users (3 from Spain, 2 from the Netherlands, and 5 from Canada) when saturation was reached. All participants acknowledged that all items were useful to evaluate the reporting of the updating process in updated clinical guidelines. Neither new items nor modifications were proposed (Table 1).

### Final Checklist

The checklist includes 16 items that can be broadly categorised into three themes: (1) presentation (e.g., clinical guideline sections and recommendations), (2) editorial independence (e.g., the working group and funding), and (3) the methodology used (e.g., search strategy and evidence synthesis) (Table 4). Those attending the presentation of the checklist workshop at the G-I-N 2015 conference reviewed and agreed with the final version of the checklist.

**Table 4:** Final version of CheckUp.

Item	Assessment	Reported on Page Number	Notes
1. The updated version can be distinguished from the previous version of the clinical guideline.	□ Yes □ No □ Unclear □ Not applicable		
2. The rationale for updating the clinical guideline is reported.	□ Yes □ No □ Unclear □ Not applicable		
3. Changes in the scope and purpose between the updated and previous version are described and justified.	□ Yes □ No □ Unclear □ Not applicable		
4. The sections reviewed in the updating process are described.	□ Yes □ No □ Unclear □ Not applicable		
5. Recommendations are clearly presented and labelled as new, modified, or not changed. Deleted recommendations are clearly noted.	□ Yes □ No □ Unclear □ Not applicable		
6. Changes in recommendations are reported and justified.	□ Yes □ No □ Unclear □ Not applicable		
7. The panel participants in the updated version are described.	□ Yes □ No □ Unclear □ Not applicable		
8. Disclosures of interests of the group responsible for the updated version are recorded.	□ Yes □ No □ Unclear □ Not applicable		
9. The role of the funding body for the updated version is identified and described.	□ Yes □ No □ Unclear □ Not applicable		
10. The methods used for searching and identifying new evidence in the updating process are described.	□ Yes □ No □ Unclear □ Not applicable		
11. The methods used for evidence selection in the updating process are described.	□ Yes □ No □ Unclear □ Not applicable		
12. The methods used to assess the quality of the included evidence in the updating process are described.	□ Yes □ No □ Unclear □ Not applicable		
13. The methods used for the evidence synthesis in the updating process are described.	□ Yes □ No □ Unclear □ Not applicable		
14. The methods used for externally reviewing the updated version are described.	□ Yes □ No □ Unclear □ Not applicable		
15. The methods and plan for implementing the changes of the updated version in practice are described.	□ Yes □ No □ Unclear □ Not applicable		
16. The plan and methods for updating the new version in the future are reported.	□ Yes □ No □ Unclear □ Not applicable		

## Discussion

We developed CheckUp through a comprehensive development process, including the use of systematic reviews, assessment of updated clinical guidelines, and engagement of the international guideline community through semistructured interviews, a Delphi consensus survey, and an external review.

### Main Findings

Across the different processes, an alignment and consensus of opinion emerged between what was documented in the literature and the expectations of clinical guideline developers, users, and researchers in regards to what information ought to be reported in an updated clinical guideline. CheckUp includes 16 items regarding the presentation of the updated clinical guideline, editorial independence, and the methodology used in the clinical guideline updating process.

CheckUp was primarily developed to evaluate the completeness of reporting in updated guidelines. Additionally, the tool can inform guideline developers about strategies for updating clinical guidelines and their reporting requirements. An explanation and elaboration article for the CheckUp is published as a supporting information article (S1 Appendix). CheckUp can be used in several ways. The checklist can provide guidance to developers who update clinical guidelines, by providing methodological principles that should be incorporated into the updating process, as well as strategies for reporting this information. The checklist can be applied by users or appraisers of clinical guidelines to assess whether updated clinical guidelines align with the CheckUp items. We suggest that a minimum of two reviewers assess the reporting of the guideline updating process independently, with the help of a third reviewer if there is a need of reaching consensus.

### Our Results in the Context of Previous Research

Updating is a crucial part of maintaining the trustworthiness of clinical guidelines [1,2]. Since clinical guidelines have a limited lifespan, updating clinical guidelines is crucial to maintain the validity of the recommendations [3,9,36,37]. Although the importance of regular updating has been recognised and clinical guidelines may have an “expiration date,” little research has been conducted in the field so far [13,15–18]. Published standards for trustworthy guidelines require the description of updating plans [1,2]; however, these standards do not provide specific guidance about the detailed reporting of the updating process of guidelines.

### Strengths and Limitations

Our CheckUp proposal has several strengths. For the development process, we systematically reviewed the evidence and followed EQUATOR and Moher’s criteria [21,22]. Also, by applying a formal consensus method (Delphi survey) and collecting experts’ opinions (semistructured interviews and external reviews), we reached a fair understanding of clinical guideline methodologists’ and users’ perceptions about the updating of clinical guidelines. Finally, there was fairly strong overall consensus during the development of CheckUp.

Our study has some limitations. We used consensus methods and convenience samples of clinical guideline stakeholders. However, across the different processes, an alignment and consensus of opinion emerged on what clinical guideline developers, users, and researchers expect to see reported in updated clinical guidelines. Another potential limitation is that CheckUp includes some items that may partially overlap with some items that are present in other instruments [19,20]; however, we think this is a minor limitation as CheckUp differs for the most part and has a very specific and differentiated goal. Finally, we did not collect potential conflicts of interest in our panel.

### Implications for Practice and Research

CheckUp can be used for multiple purposes. Firstly, guideline developers can use it both for the reporting of their guidelines and to plan their updating processes. Guideline users can assess the reporting of updated guidelines. Editors may request its completion from guideline authors. CheckUp provides an overall picture of how complete the updating process is reported in updated clinical guidelines. Being a reporting checklist, CheckUp does not evaluate the quality of the updating processes, as there are no gold standards for this process. Currently, the G-I-N Updating Guidelines Working Group (http://www.g-i-n.net/working-groups/updating-guidelines) is undertaking an analysis of current guideline updating methods worldwide. From this work, strategies or advice might come on how we might assess guideline updating quality.

There are currently no gold standards for guideline updating methodology. Nonetheless, updating is key to ensuring trustworthy, implementable, and clinically relevant recommendations. Current guideline evaluation tools or guideline method resources (e.g., AGREE II, Grading of Recommendations Assessment, Development, and Evaluation (GRADE), IOM Standards, and the like) are not simply transferable to the conceptual requirements of an updated guideline. CheckUp addresses the gap: it has been supported by our study participants and is a resource that complements (rather than competes with) the other high-quality tools available in the guideline enterprise.

Further rigorous research in updating clinical guidelines is warranted, and we invite users to comment on the items and the usability of CheckUp. It would be important to assess the impact of CheckUp in the updating clinical guideline field over the next few years [16]. When dynamic or living guidelines become a reality, [38] some adaptation of CheckUp could potentially be necessary. Finally, the G-I-N Updating Guidelines Working Group will continue to play a key role in this work and in moving forward the updating agenda in the clinical guideline enterprise.

## Supporting Information

S1 Appendix CheckUp: Explanation and elaboration of a checklist for the reporting of updating clinical guidelines. (DOCX)

## Abbreviations

AGREE: Appraisal of Guidelines for Research and Evaluation
CheckUp: Checklist for the Reporting of Updated Guidelines
EQUATOR: Enhancing the Quality and Transparency of Health Research
G-I-N: Guidelines International Network
GRADE: Grading of Recommendations Assessment, Development, and Evaluation
IOM: Institute of Medicine
