# Supplementary material for: Reporting Items for Updated Clinical Guidelines: Checklist for the Reporting of Updated Guidelines (CheckUp)
Source: PLoS Med. 2017 Jan 10;14(1):e1002207. doi: 10.1371/journal.pmed.1002207 (PMC5224740; doi:10.1371/journal.pmed.1002207)
Supplement: S1 Appendix — (DOCX) [file pmed.1002207.s001.docx]

# CheckUp: Explanation and elaboration of a checklist for the reporting of updating clinical guidelines

Vernooij RWM^1^, Martínez García L^1^*, Brouwers M^2^, Alonso-Coello P^1,2^; CheckUp Panel.

1. Iberoamerican Cochrane Centre, and Biomedical Research Institute Sant Pau (IIB Sant Pau), Barcelona, Spain.

2. CIBER of Epidemiology and Public Health (CIBERESP), Spain.

3. Program in Evidence-based Care, Cancer Care Ontario, Canada.

Membership of the UpCheck Panel is provided in the Acknowledgements.

***** laura.martinez.garcia@cochrane.es

# Abstract

## Background

Due to the continuous emergence of new evidence, clinical guidelines have a limited life-time. However, evidence based reporting standards for the updating of clinical guidelines are still not available. In this article we present CheckUp (CHECKlist for the reporting of UPdating clinical guidelines) and provide explanations, and examples for each item. In a previous article we present the methodology to develop CheckUp.

## Methods and findings

We developed an initial list of items – including explanation and examples – based on an overview of research on clinical guideline updating, the AGREE (Appraisal of Guidelines for Research and Evaluation) II Instrument, and the advice of the CheckUp Panel. A multi-step process was used to refine this list including an assessment of existing guidelines, a Delphi consensus survey, key informant interviews, and a formal external review by both clinical guideline methodologists and users. CheckUp includes sixteen items that address the: 1) presentation (version, rationale for updating, changes in scope and purpose, reviewed sections, recommendation labels, changes in the recommendations); 2) editorial independence (panel participants and disclosures of interest); and 3) methodology of the updating process (methods for search strategy, evidence selection, quality assessment, evidence synthesis, external review, implementation, and future updates).

## Conclusions

CheckUp can be used to evaluate the completeness of reporting in updated guidelines, and as a tool to inform guideline developers about reporting requirements. This explanation and elaboration article aims to facilitate the use of CheckUp both, for clinical guidelines users and developers, by explaining the rationale of the items, and providing relevant examples of adequate reporting.

# Keywords

Evidence-Based Medicine/Standards, Practice Guidelines as Topic, Information Dissemination, Publishing/Standards, Quality Control, Terminology as topic, Program Development, Program Evaluation

# Word count

Abstract: 255 words

Manuscript: 5186 words.

# Background

Trustworthy clinical guidelines aim to assist decision making by providing recommendations that are informed by the best available evidence, and include an assessment of the benefits and harms of alternative care options [1,2]. Due to the continuous emergence of new research evidence (i.e. changes in available interventions, effects, or costs) [3], appropriate updating to maintain the trustworthiness of clinical guidelines is challenging since it requires regular reviewing [4,5]. To date, little attention has been paid to the methodology for updating clinical guidelines [5-8].

Very little guidance in reporting standards regarding the updating process of clinical guidelines has been identified [9,10]. To address this need, in a partnership of the Iberoamerican Cochrane Center [www.cochrane.org], the AGREE Collaboration [www.agreetrust.org] and the Guidelines International Network (G-I- N) Updating Working Group [www.g-i-n.net/working-groups/updating-guidelines], we have developed CheckUp (CHECKlist for the reporting of UPdated guidelines). The aim of CheckUp is to evaluate the completeness of reporting the updating process in clinical guidelines, and to inform clinical guideline developers about reporting requirem**e**nts.

This series of two articles about CheckUp is targeted at guideline developers and users of guidelines. In the first article we presented the methodology of the development process [11]. In this second article we explain and elaborate on all checklist items including the explanation and examples with the goal to facilitate the use of CheckUp.

# Methods

The development of CheckUp consisted of four phases: 1) panel selection; 2) generation of the initial checklist; 3) optimisation of the checklist; and 4) approval of the final checklist. We reported the detailed methodology of the development process of CheckUp in a previous article [11].

To advise on the development of CheckUp, a panel was convened comprised of individuals with expertise in clinical guideline development, updating and/or research methodology. The purpose of the panel was to provide expert advice on the checklist and to participate in a Delphi survey. A core group of authors (RWMV, LMG, MB, and PAC) was established to provide time-sensitive and operational advice.

We developed an initial list through discussion and brainstorming, taking into account the following aspects: 1) key research literature, including two systematic reviews about updating [7,8,10]; 2) the AGREE II instrument [12]; and 3) the CheckUp Panel experience. In this process the initial list of items, examples, and explanations were generated. We refined the checklist subsequently by a multi-step process that included: 1) an assessment of a sample of updated clinical guidelines; 2) semi-structured interviews; 3) a Delphi consensus survey; and 4) an external review with clinical guideline methodologists and users. Finally, all CheckUp Panel members reviewed and approved the final version of the checklist (Table 1).

## ***Table 1. CheckUp (Checklist for reporting the Updating process) items and assessment***

| **Item** | **Assessment** | **Reported on page number.** | **Notes** |
| --- | --- | --- | --- |
| 1. The updated version can be distinguished from the previous version of the clinical guideline | - Yes - No - Unclear - Not applicable |  |  |
| 1. The rationale for updating the clinical guideline is reported | - Yes - No - Unclear - Not applicable |  |  |
| 1. Changes in the scope and purpose between the updated and previous version are described and justified | - Yes - No - Unclear - Not applicable |  |  |
| 1. The sections reviewed in the updating process are described | - Yes - No - Unclear - Not applicable |  |  |
| 1. Recommendations are clearly presented and labelled as new, modified, or not changed. Deleted recommendations are clearly noted | - Yes - No - Unclear - Not applicable |  |  |
| 1. Changes in recommendations are reported and justified | - Yes - No - Unclear - Not applicable |  |  |
| 1. The panel participants in the updated version are described | - Yes - No - Unclear - Not applicable |  |  |
| 1. Disclosures of interests of the group responsible for the updated version are recorded | - Yes - No - Unclear - Not applicable |  |  |
| 1. The role of the funding body for the updated version is identified and described | - Yes - No - Unclear - Not applicable |  |  |
| 1. The methods used for searching and identifying new evidence in the updating process are described | - Yes - No - Unclear - Not applicable |  |  |
| 1. The methods used for evidence selection in the updating process are described | - Yes - No - Unclear - Not applicable |  |  |
| 1. The methods used to assess the quality of the included evidence in the updating process are described | - Yes - No - Unclear - Not applicable |  |  |
| 1. The methods used for the evidence synthesis in the updating process are described | - Yes - No - Unclear - Not applicable |  |  |
| 1. The methods used for externally reviewing the updated version are described | - Yes - No - Unclear - Not applicable |  |  |
| 1. The methods and plan for implementing the changes of the updated version in practice are described | - Yes - No - Unclear - Not applicable |  |  |
| 1. The plan and methods for updating the new version in the future are reported | - Yes - No - Unclear - Not applicable |  |  |

# Explanation and Elaboration of the items and examples of CheckUp

The answer possibilities for all items are: 1) Yes, 2) No, 3) Unclear, and 4) Not applicable. Whenever the users of CheckUp believe that certain concepts are not applicable in the updated guideline subject to assessment, the answer “Not applicable” can be used. Consequently, the denominator of total items should be adjusted. Similarly, the answer possibility “Unclear” can be used whenever the user is doubting whether the items is adequately described in the updated clinical guideline.

## Item 1. The updated version can be distinguished from the previous version of the clinical guideline

### Explanation

The clinical guideline users must be able to easily identify that they are consulting an updated version of the clinical guideline. If possible, a reference to the previous version should be provided and the clinical guideline has to be identified as an update.

### Examples

- Infection prevention and control guideline for cystic fibrosis: 2013 update [13].
- This is an updated version of the clinical guideline previously published in 2014 (illustrative example).

In these examples, branding the updated version has been achieved by using the term “update” and the publication date (year) in the title. Other options include new version number/code in the title.

## Item 2. The rationale for updating the clinical guideline is reported

### Explanation

There are different situations that might trigger the updating process of a clinical guideline (e.g. elapsed time frame or identification of new evidence). Clinical guideline users should be able to identify these reasons. Whenever the rationale for updating differs among various sections in the clinical guideline, these differences should be reported.

### Examples

- After publishing the clinical guideline in 2006, a time period of five years for starting the updating process was considered adequate. The updating process was therefore started in 2011. After review and analysis of the new evidence since the previous version of the clinical guideline, the Update Panel concluded that the new evidence was compelling enough to warrant substantive changes in various recommendations (illustrative example).
- Recently, three new randomised controlled trials have been published.Given the importance of these studies and their relevance to this clinical guideline, an update of this clinical guideline was undertaken by the Update Panel. This new evidence prompted a new systematic review of the literature (illustrative example).
- In the yearly revision of all the clinical guidelines with the clinical experts included in our working group, two members indicated the existence of two new randomised controlled trials that are not included in the previous version of this clinical guideline. Therefore, we decided to update this clinical guideline and conduct a new systematic review of the literature (illustrative example).

In these examples different rationales for updating a clinical guideline have been stated and other reasons are imaginable: 1) a certain pre-specified time frame elapsed; 2) new evidence is expected or known to exist; or, 3) alerts / clinical experts indicate a possible obsolescence of the recommendations in the current clinical guideline.

## Item 3. Changes in the scope and purpose between the updated and previous version are described and justified

### Explanation

If in the updating process the scope and purpose of the clinical guideline change, the clinical guideline users have to be able to identify the differences in the scope and purpose of the updated version in contrast with the previous version, including the justification for these changes. A statement should be included if there are no differences in the scope and purpose between the updated and the previous version of the clinical guideline.

### Examples

- The scope of the clinical guideline changed since the working group decided to expand due to new identified evidence on the prevention of Urinary Tract Infections (UTIs) in patients treated with catheters, which was not included in the previous version of the clinical guideline. Consequently, the scope of the clinical guideline is extended to the prevention, diagnosis, and treatment of UTIs in patients treated with catheters (illustrative example).
- Given the availability of randomised controlled trials included in the updated version of this clinical guideline, the scope of the inclusion criteria was modified to only include higher level evidence. Consequently, the scope of the clinical guideline changed to give insights in the prevention, diagnosis, and treatment of schizophrenia in primary care setting, based only on the highest level evidence available. Case controlled studies included in the previous version were moved to appendix X and serve now as historical evidence (illustrative example).

In these examples, reasons for changes in the scope and purpose of the clinical guideline have been included, by providing modified or new clinical questions. Other plausible reasons for changes in the scope and purpose are modifications to the target population, interventions, comparisons or outcomes, or a new clinical setting.

## Item 4. The sections reviewed in the updating process are described

### Explanation

An updated clinical guideline may be modified in all, some, or no sections (chapters, questions, etc.) compared to the previous version of the clinical guideline. Which sections have changed, which sections were reviewed but remained unchanged, and, if applicable, which sections have not changed should be transparently reported.

### Examples

- Summary of updates, by section of the clinical guideline (Table 2) (illustrative example).

In this example, an overview has been provided with information about which sections have been, for example, reviewed and modified, reviewed but not modified, or not been reviewed. The terminology is not mandatory, it might be adapted to the needs of each organisation.

## Table 2. Example for Item 4

| **Clinical Guideline Sections** | **Actions** |
| --- | --- |
| 1. Key recommendations | New section |
| 1. Diagnosis of bacterial UTI in patients with catheters | Not reviewed |
| 1. Management of bacterial UTI in adult women | Reviewed and modified |
| 1. Management of bacterial UTI in pregnant women | Reviewed and modified |
| 1. Management of bacterial UTI in adult men | Reviewed but not modified |
| 1. Management of bacterial UTI in patients with catheters | Reviewed and modified |
| 1. Management of bacterial UTI in new-borns | Deleted |
| 1. Prevention of bacterial UTI in patients with catheters | New section |
| 1. Provision of information | Reviewed and modified |
| 1. Implementing the clinical guideline | Reviewed but not modified. |
| 1. External review | Not repeated |

Abbreviation: UTI: Urinary Tract Infection.

## Item 5. Recommendations are clearly presented and labelled as new, modified, or not changed. Deleted recommendations are clearly noted

### Explanation

If some recommendations change due to newly identified evidence, the clinical guideline users should be able to identify these changes at the recommendation level. Recommendations can be labelled as new, modified, or not changed. In case recommendations are deleted, clinical guideline users should be alerted.

### Examples

- Discuss with people who have or who are at risk of breast-cancer related lymphoedema that there is no indication that exercise prevents, causes or worsens lymphoedema [new 2014] [14].
- Explore whether the patient experienced in the past allergies to any antibiotics that can be used in the treatment of UTIs [reviewed and not changed 2014] (illustrative example).
- Due to the fact that a new and more effective antibiotic Y for the treatment of UTIs is available since 2013, we decided to delete three recommendations that previously advocated the use of antibiotic X. These deleted recommendations have been included in appendix X (illustrative example).
- Overview of recommendations included in section X (Table 3) (illustrative example).

## Table 3. Example for Item 5

| **Recommendations** | **Status** |
| --- | --- |
| 1. Explore whether the patient experienced in the past allergies to any antibiotics that can be used in the treatment of UTIs. | Reviewed and not changed 2014 |
| 1. Explore whether the patients is using at this current moment any other antibiotics for the treatment of any other infection. | Not reviewed |

In these examples, labels for the status of recommendations have been used, including [new 2014], whenever a recommendation is newly implemented in the updated version and was not included in the previous version. Other possible terminology for labelling recommendations could be: "[modified 2014]" if the recommendation has been reviewed and modified in 2014; "[2009]" if the recommendation has not been reviewed since the previous version of the clinical guideline; or in the case of reviewed but unchanged recommendations, a label like "[reviewed and not changed 2014]" might be used. An overview with all the recommendations and their corresponding labels can also be provided. If applicable, an alert about the deleted recommendations should be included.

## Item 6. Changes in recommendations are reported and justified

### Explanation

In the updated version, clinical guideline users should be able to identify the changes made in the recommendations. Information regarding which specific parts of the recommendations have been changed, including a justification of these changes, should be provided. To avoid possible confusion between updated and previous recommendations this information should be included in an appendix. A statement should be included if there are no differences between the recommendations of the updated and previous version of the clinical guideline.

### Examples

- Appendix: Overview of the new recommendation, previous recommendations, and the rationale for changes (Table 4) [15].

In this example an examination has been given to compare the previous recommendation and the recommendation after the update. To illustrate the rationale for updating the recommendations, a reflection about the evidence inducing the changes has been given.

## Table 4. Example for Item 6

| **New recommendation** | **Previous recommendation** | **Reason for change/deletion** |
| --- | --- | --- |
| For people with unexplained infertility, mild endometriosis or ‘mild male factor infertility’, who are having regular unprotected sexual intercourse:   - do not routinely offer intrauterine insemination, either with or without ovarian stimulation (exceptional circumstances include, for example, when people have social, cultural or religious objections to IVF) - advise them to try to conceive for a total of 2 years (this can include up to 1 year before their fertility investigations) before IVF will be considered. [new 2013] | Where intrauterine insemination is used to manage male factor fertility problems, ovarian stimulation should not be offered because it is no more clinically effective than unstimulated intrauterine insemination and it carries a risk of multiple pregnancy. [A] | New evidence has shown that IUI with or without stimulation is no more effective than no treatment. Therefore, it is no longer recommended. |

## Item 7. The panel participants in the updated version are described

### Explanation

This item refers to the panellists who were involved in the updating process. It is plausible that in the time period between the previous and the updated version the composition of the clinical guideline panel changes, especially when the scope and purpose of the clinical guidelines vary. Additionally, the panel participants of the previous version of the clinical guideline should be reported, especially in the case of recommendations that were not changed in the updating process. Patients, public, and other stakeholders' involvement in the panel responsible for updating the clinical guideline should be clearly reported.

### Examples

- The panel members involved in *the 2014* update (including their names, disciplines, organisation, and the role of each panellist) are listed in appendix 1. The composition of the group responsible for the previous version of the clinical guideline is included in this appendix (illustrative example).

In this example, the name, expertise, affiliated organisations, geographical location, the role of each member of the panel responsible for updating the clinical guideline, and of those responsible for the previous version of clinical guideline are clearly described.

## Item 8. Disclosures of interests of the group responsible for the updated version are recorded

### Explanation

All group members who participated in the updating process should disclosure their conflicts of interest (CoI) before starting the updating process. The CoI of those involved in the updating process are important for the recommendations that have been updated or newly implemented. Additionally, the CoI of the participants who were involved in the previous version and not in the updating process should still be reported, since they remain relevant for the recommendations that are not reviewed or those that have not been changed.

### Examples

- Members of the Update Panel completed a CoI form, which requires disclosure of financial and other interests that are relevant to the subject matter of the clinical guideline, including relationships with commercial entities that are reasonably likely to experience direct regulatory or commercial impact with the implementation of the clinical guideline. These disclosure forms are included on the website [link] (illustrative example).
- In accordance with the procedures, the majority of members of the Update Panel did not disclose any such relationship. For the recommendations regarding medicine X, one participant reported substantial CoI, due to previous research funding and the involvement in publications regarding this medicine. Consequently, this participant has been excluded from the discussions about medicine X (illustrative example).
- In addition, the CoI of the panel responsible for the development process of the previous version of the clinical guideline are included in appendix VI. These CoI are still valid for the recommendations that are not reviewed (illustrative example).
- Measures for handling intellectual CoI, such as including published work of those responsible for constructing the recommendation, have been reported in appendix VI (illustrative example).

In these examples, a description of the CoI, type and in what form, how these were sought, and how these were addressed, has been included. The CoI of the people involved solely in the updating process, or in both the updating process and the development process of the previous version, or only in the development process of the previous version, have been described.

## Item 9. The role of the funding body for the updated version is identified and described

### Explanation

Clinical guidelines are frequently developed with the help of an external funding body, in the form of financial contribution used for realising the updating process. A description of the role of the funding body should be reported in the updated version, including an indication of the amount of funds provided. If a clinical guideline is self-funded (with no external support) this should be explicitly reported. If the funding for the updating process differs from the funding of the previous version, a description of both funding bodies should be given.

### Examples

- Support for this clinical guideline was provided by the Society for Healthcare Epidemiology of America (SHEA) and the Infectious Diseases Society of America (IDSA). The financial support of the previous version of this clinical guideline solely consists of the Society for Healthcare Epidemiology of America. Both organisations responsible for the external funding did not have any influence in the content of the clinical guideline [16].

In this example, an explicit statement is included in the updated version, providing details of the funding body. Further details on the amount of funds provided, the role of the funding body in the updating process, and its influence on the content of the clinical guideline are reported.

## Item 10. The methods used for searching and identifying new evidence in the updating process are described

### Explanation

A complete documentation of the search strategy should be included in the updated version to allow clinical guideline users to reiterate the search strategy, to be informed about the searched sources, and to evaluate the quality of the search strategy. Differences in the search strategy between the updated and the previous version should be justified. In addition, whenever different search strategies have been used for different recommendations or clinical questions, this should be stated.

### Examples

- Electronic databases (MEDLINE and EMBASE) were searched from 2000 to 2008. The single exception was for question 7, where it was stipulated in the research question to exclude studies published before 2003. The full search strategy is described in appendix II [17].
- With the exception of the searched interval dates, the search strategy followed the previous strategy used in the preceding clinical guideline (appendix II): MEDLINE, EMBASE, and CINAHL: September 2010 to November 2014; Cochrane library: 2014 (illustrative example).

In these examples, details of the used search strategy for identifying the new evidence in the updated version are reported, including the search terms used for all recommendations and clinical questions (appendix), consulted databases (e.g. MEDLINE, EMBASE), and the time period considered. The complete search strategy is included in an appendix. Finally, the differences in methodology of the search strategy between the updated and the previous version are reported.

## Item 11. The methods used for evidence selection in the updating process are described

### Explanation

Criteria for including or excluding the new identified evidence in the updating process should be explicitly described. If the inclusion and exclusion criteria in the updated version differ from those used in the previous version, these changes should be reported and justified. A statement should be included if there are no differences in methodology for evidence selection between the updated and the previous version.

### Examples

- The inclusion and exclusion criteria differ from those used in the 2009 clinical guideline. For the updated version, we included prevention of UTI for patients treated with catheters. Therefore, the inclusion criteria are now: systematic reviews or randomised controlled trials published in English; addressing the prevention, diagnosis, or treatment of UTIs for patients treated with catheters (illustrative example).
- We applied the same eligibility criteria (inclusion and exclusion criteria) as in the previous clinical guideline. See appendix III (illustrative example).

In these examples, the inclusion and exclusion criteria used are clearly reported. These criteria refer to study design, languages, and study objectives. In addition, in the inclusion criteria reference has been made to the clinical question of the clinical guideline.

## Item 12. The methods used to assess the quality of the included evidence in the updating process are described

### Explanation

A complete documentation of the evidence assessment allows clinical guideline users to examine the rating of the quality of the evidence. The differences in methodology used to assess the evidence between the updated and the previous version should be reported, including the rationale behind those differences. Additionally, whenever the methodology (e.g. instrument or tool) is used in the updated version differs from that used in the previous version it should be described whether the evidence included in the previous version was also assessed with the new methodology. A statement should be included if there are no differences in the methodology for evidence assessment between the updated and previous version.

### Examples

- In the updating process of the clinical guideline, the quality assessment was conducted with the GRADE (Grading of Recommendations Assessment, Development and Evaluation) system. In the GRADE approach, the quality of the evidence is obtained by taking into consideration several factors. An assessment of the risk of bias, inconsistency, indirectness, imprecision, and other considerations (including publication bias) has been conducted. We have used GRADE to evaluate the whole evidence base, not just the new studies identified (illustrative example).
- The methods used to assess the quality of the studies found during the updating were similar as those used in the previous version of the clinical guideline (illustrative example).

In these examples, the methodology used to assess the risk of bias and/or the quality of the evidence is described (e.g. GRADE methodology or other instruments), as well as the differences with the previous versions.

## Item 13. The methods used for the evidence synthesis in the updating process are described

### Explanation

The methodology used to combine multiple sources of quantitative and/or qualitative evidence should be reported in the updated version. Furthermore, the methodology used to translate the evidence into recommendations should be stated in the updated version. A statement should be included if there are no differences in methodology for evidence synthesis between the updated and the previous version.

### Examples

- Generated summaries of the evidence by outcome: 1) randomised studies: meta-analysed, where appropriate and reported in GRADE profiles for clinical studies. 2) Observational studies: data presented as a range of values in GRADE profiles. 3) Qualitative studies: each study summarised in a table (available in an appendix) where possible, and the quality of included studies assessed against the NICE quality checklists for qualitative studies. Key common themes between studies which were relevant to the review question were summarised and presented with a comment of the quality of studies contributing to the themes in the main clinical guideline document. GRADE does not have a system for rating the quality of evidence for qualitative studies or surveys, and therefore there are no GRADE quality ratings for the themes identified [18].
- The methods for evidence synthesis during the updating process followed those used in the development of the previous version of the clinical guideline (illustrative example).
- Studies in the updated and previous version were not meta-analysed due to the paucity of evidence reporting individual outcomes. GRADE evidence profiles have, therefore, been presented in the appendix XI, with the new evidence clearly identified (illustrative example).

In these examples, the methodology used for synthesising the newly identified evidence (quantitative and/or qualitative) with the evidence included in the previous version is reported.

## Item 14. The methods used for externally reviewing the updated version are described

### Explanation

An updated version should be externally reviewed by experts in the clinical area, methodological experts, and/or public consultation. The reviewers should not have been involved in the updating process of the clinical guideline. On the other hand, if no changes have been made, an external review of the updated version might seem less appropriate. Where external review was deemed appropriate, the methods, results and impact should be described, including information of the reviewers.

### Examples

- The updated version was reviewed in draft form by the following expert referees, who were members of the previous clinical guideline development group. All expert referees made declarations of interest and further details of these are available on request from the SIGN Executive [18].
- Given that the recommendations were not changed, an external review of the updated version was not undertaken (illustrative example).
- Given that solely minor changes were made to the recommendations, a modified strategy of the external review process was undertaken. Specifically, we only asked the group participating in the external review to provide comments on the recommendations that have been changed (illustrative example).

In these examples, documentation of the methodology used to conduct the external review has been presented. If an external review of the updated version has been conducted, it is stated whether specific instructions to appraise the updated version were provided to the reviewers.

## Item 15. The methods and plan for implementing the changes of the updated version in practice are described

### Explanation

If applicable, specific accompanying materials produced to support the implementation of the updated version should be provided. The implementation plan should lay emphasis on the new recommendations of the updated version, or on the recommendations with significant changes. In addition, the implementation plan from the previous version might have been evaluated, and consequently improved for the updated version. A statement should be included if there are no differences in methodology for the implementation plans or strategies between the updated and the previous version.

### Examples

- - The implementation plan of the updated version includes the following aspects: 1) Regular audit, with feedback of non-adherence to local clinical guidelines (including specific clinician feedback). This should be actively discussed and acted upon on a regular basis. 2) Active involvement and support from local senior staff or respected opinion leaders for the implementation strategy programme. 3) Indicate the changes introduced in recommendations in the updated version with respect to the previous version, by directing clinical guideline users to appendix X (illustrative example).
  - The methods for the implementation plan during the updating procedure followed those used in the development of the previous version (illustrative example).

In these examples the methodology and the specific aspects of the implementation plan of the updated clinical guideline have been given. If this consists of different aspects, they have all been illustrated.

## Item 16. The plan and methods for updating the new version in the future are reported

### Explanation

Updating is a crucial process for maintaining the validity of recommendations. A clear statement about the methodology and the plan of the forthcoming updating procedures should be provided. It should be clearly noted whenever the updating process will differ among clinical questions or recommendations. Additionally, if specific cases exist that trigger an update before the time frame, these should be reported. If deemed appropriate, a specific time period for the next update, including a rationale for this period, must be provided.

### Examples

- This clinical guideline was issued in 2014 and will be considered for review within two years to assure the validity of this clinical guideline because of the relatively quick advances in this clinical area. The panel responsible for updating the clinical guideline in 2014 will be contacted again for participation in the updating process in 2016. Any updates to the clinical guideline in the interim period will be noted on the website. In principle, the same methodology for this current update will be used for the update in 2016 (illustrative example).

In this example, the procedure, including the time interval, for updating the clinical guideline in the future is provided in the updated version, including an established panel that will be responsible for updating the clinical guideline.

# Discussion

We developed CheckUp using a rigorous development process, including the use of systematic reviews, assessment of updated clinical guidelines, and engagement of a large international clinical guideline community through semi-structured interviews, a Delphi consensus survey, and a wide external review process. CheckUp includes 16 items regarding the presentation, the editorial independence, and the methodology used in the clinical guideline updating process.

Like similar explanation and elaboration documents from other instruments [19-25] the primary aim of this manuscript is to outline a framework for adequate use of the CheckUp checklist. With this article we aim to improve its usability by explaining the rationale of the items, and by providing relevant examples of optimal reporting.

### Our results in the context of previous research

The methodology for updating clinical guidelines is not standardised and should be more rigorous [7]. This lack of rigour, compared to de novo development of clinical guidelines, might be due to different reasons including: a) the scarcity of methodological research on the updating process [8]; b) lack of guidance in handbooks of clinical guideline institutions [9,10]; c) a clear emphasis on developing rather than updating in most organisations over the last two decades [5].

In CheckUp, the format of updated clinical guidelines, recommendations, and updated sections is covered in items 1 to 6. Nowadays, there is a gap in knowledge regarding the presentation formats of updated clinical guidelines. An analysis of clinical guidelines updated by the National Institute of Care Excellence (NICE), a key organisation in the clinical guideline area, observed concerning variability in the presentation formats of the recommendations’ changes, and a lack of justification for those changes [26]. The DECIDE (Developing and Evaluating Communication Strategies to Support Informed Decisions and Practice Based on Evidence; http://www.decide-collaboration.eu/) project, an initiative by the GRADE working group, has developed several presentation formats for recommendations of clinical guidelines [27,28]. However, the project did not address the presentation of updated recommendations.

A description of the updating panel, the disclosures of interest, and the role of the funding body are covered in items 7 to 9. Both, the role in the working group and disciplines of the various members of the panel, responsible for updating a clinical guideline need to be carefully taken into account [29]. Similarly, the conflicts of interest of the members of the original clinical guideline should be regularly reviewed throughout the process of monitoring and updating. [30].

In CheckUp, the methodology of the updating process is covered in items 10 to 16. Updating of a clinical guideline should be performed with a similar degree of rigour and explicitness as in the development of a de novo clinical guideline. However, in practice updating clinical guidelines seems to be methodologically poorer conducted than developing de novo clinical guidelines [31].

Although the checklist provides methodological principles on updating and reporting, it does not advocate a single operation strategy for updating a clinical guideline. There is no evidence for a “gold standard”, there are is little research evidence in the field, and often the original de novo methodology is used during the updating process [8]. In response to this gap, CheckUp is a first step, determining what content has to be reported in updated clinical guidelines.

### Strengths and limitations

We believe that the strength of our study concerns the elaborate development process of the CheckUp. For the development process we followed the criteria of the EQUATOR (Enhancing the QUAlity and Transparency Of health Research) network and included the recommendations stated by Moher et al (2011) [32,33]. In the development process of CheckUp we used several methods to ensure inclusion of developers´ and users´ input internationally. By applying a formal consensus method (Delphi survey) and collecting experts’ opinions using diverse methods (semi-structured interviews and external reviews), we reached a fair understanding of clinical guideline methodologists’ and users’ perceptions of the updating of clinical guidelines. Complemented by empirical evidence from the literature and an assessment of updated clinical guidelines, we believe that CheckUp includes all relevant aspects of updating clinical guidelines.

CheckUp has some limitations. One is the use of consensus methods with convenience samples of clinical guideline stakeholders. There is, hence, a potential bias with our sampling frames. However, across the different processes, an alignment and consensus of opinion emerged on what clinical guideline developers, users, and researchers expect to see reported in an updated clinical guideline. Additionally, for all items we tried to present optimal presentation formats examples from published updated clinical guidelines. However, we were not able to find for some items real examples of adequate presentation formats. Therefore, illustrative examples of what we believe to be realistic and adequate reporting have been included. However, our use of a particular example does not imply that the terminology used is mandatory.

### Implications for practice and research

CheckUp is a checklist for users and clinical guideline appraisers to assess the reporting of the updating process in updated clinical guidelines. Besides, CheckUp can be used by clinical guideline developers as a helping checklist for planning and conducting the reporting of clinical guidelines that need to be updated. The checklist can, hence, provide guidance to developers who are updating clinical guidelines through methodological principles that should be incorporated into the clinical guideline updating process, and strategies for reporting the clinical guideline.

CheckUp will be updated in the near future whenever real examples of adequate reporting will be identified. We advise the updating panels that are responsible for updating clinical guidelines to take the items of CheckUp into account. Additionally, there is a need to evaluate the reporting of updated guidelines with CheckUp. It would be desirable to also assess the impact of this checklist in the clinical guideline field over the next few years. The Guidelines International Network (G-I-N) Updating Working Group [www.g-i-n.net/working-groups/updating-guidelines] will be playing a crucial role in this work and in moving forward the updating agenda in the clinical guideline enterprise.

In addition, like every resource designed to improve evidence-based methodology, we recognize that CheckUp is the first iteration of the checklist. As specific methods for updating continue to best tested and evolve, and standard practices emerge, these innovations will be reflected in future revisions of CheckUp.

# Acknowledgements

Vernooij RWM, Alonso-Coello P, Brouwers M, Martínez García L, Florez ID, Iorio A, James R, Sanabria AJ, Selva A, Shekelle PG, and Vandvik PO are members of the G-I-N Updating Guidelines Working Group. The members of the CheckUp panel are: Vernooij RWM, Alonso-Coello P, Brouwers M, Martínez García L, Ada L, Alemán A, Arévalo-Rodriguez I, Becker M, Burgers JS, Chan W, Delvaux N, Duggan G, Enciso Olivera CO, Etxeandia-Ikobaltzeta I, Florez ID, Follmann M, Gartlehner G, Iorio A, James R, Jones SL, Kotzeva A, Lloyd M, López Gallegos D, Louro-González A, Marin Leon I, Martí-Carvajal A, Meerpohl JJ, Pardo R, Rojas-Reyes MX, Rotaeche R, Sanabria AJ, Selva A, Shekelle PG, Sierra Matamoros FA, van de Velde S, Vandvik PO, Willett S.

Robin W.M. Vernooij is a doctoral candidate at the Paediatrics, Obstetrics and Gynaecology and Preventive Medicine Department, Universitat Autònoma de Barcelona, Barcelona, Spain. The authors thank Sandra Pequeño Saco (Iberoamerican Cochrane Centre) for her support in assessing updated clinical guidelines. The authors also thank María Victoria Leo for her help editing the manuscript.

# References

1. Laine C, Taichman DB, Mulrow C. Trustworthy clinical guidelines. Ann Intern Med. 2011;154(11): 774-775.
2. Qaseem A, Forland F, Macbeth F, Ollenschläger G, Phillips S, van der Wees P, et al. Guidelines International Network: toward international standards for clinical practice guidelines. Ann Intern Med. 2012;156(7):525-31.
3. Shekelle PG, Ortiz E, Rhodes S, Morton SC, Eccles MP, Grimshaw JM, Woolf SH. Validity of the Agency for Healthcare Research and Quality clinical practice guidelines: how quickly do guidelines become outdated? JAMA. 2001;286(12):1461-7.
4. Grupo de trabajo sobre actualización de GPC. Actualización de Guías de Práctica Clínica en el Sistema Nacional de Salud. Manual Metodológico. Plan de Calidad para el Sistema Nacional de Salud del Ministerio de Sanidad y Política Social. Instituto Aragonés dológico. de Ciencias de la Salud-I+CS; 2009. Guías de Práctica Clínica en el SNS: I+CS Nº 2007/02-01
5. Shekelle PG. Updating practice guidelines. JAMA. 2014;311(20):2072-3.
6. Burgers JS, Cluzeau FA, Hanna SE, Hunt C, Grol R. Characteristics of high-quality guidelines: evaluation of 86 clinical guidelines developed in ten European countries and Canada. Int J Technol Assess Health Care. 2003;19(1):148-57.
7. Alonso-Coello P, Martínez García L, Carrasco Gimeno JM, Solà I, Qureshi S, Burgers JS; Updating Guidelines Working Group. The updating of clinical practice guidelines: insights from an international survey. Implement Sci 2011, 6:107.
8. Martínez García L, Arévalo-Rodríguez I, Solà I, Haynes RB, Vandvik PO, Alonso-Coello P; Updating Guidelines Working Group. Strategies for monitoring and updating clinical practice guidelines: a systematic review. Implement Sci 2012, 7(1):109.
9. Becker M, Neugebauer EA, Eikermann M. Partial updating of clinical practice guidelines often makes more sense than full updating: a systematic review on methods and the development of an updating procedure. J Clin Epidemiol. 2014;67(1):33-45.
10. Vernooij RW, Sanabria AJ, Solà I, Alonso-Coello P, Martínez García L. Guidance for updating clinical practice guidelines: a systematic review of methodological handbooks. Implement Sci. 2014;9:3.
11. Vernooij RWM, Alonso-Coello P, Brouwer MC, Martínez García L; CheckUp Panel. CheckUp: reporting items for updated clinical guidelines. Submitted.
12. Brouwers MC, Kho ME, Browman GP, Burgers JS, Cluzeau F, Feder G, Fervers B, Graham ID, Grimshaw J, Hanna SE, Littlejohns P, Makarski J, Zitzelsberger L; AGREE Next Steps Consortium. AGREE II: advancing guideline development, reporting and evaluation in health care. CMAJ. 2010;182(18):E839-42.
13. Saiman L, Siegel JD, LiPuma JJ, Brown RF, Bryson EA, Chambers MJ, Downer VS, Fliege J, Hazle LA, Jain M, Marshall BC, O'Malley C, Pattee SR, Potter-Bynoe G, Reid S, Robinson KA, Sabadosa KA, Schmidt HJ, Tullis E, Webber J, Weber DJ. Infection prevention and control guideline for cystic fibrosis: 2013 update. Infect Control Hosp Epidemiol. 2014;35:Suppl 1:S1-S67.
14. NICE (2014) Advanced breast cancer (update. Diagnosis and treatment NICE clinical guideline 81). Available at https://www.nice.org.uk/Guidance/CG81 [NICE guideline].
15. [NICE (2013) Fertility: Assessment and treatment for people with fertility problems. Available at https://www.nice.org.uk/guidance/CG156 [NICE guideline]
16. Cohen SH, Gerding DN, Johnson S, Kelly CP, Loo VG, McDonald LC, Pepin J, Wilcox MH; Society for Healthcare Epidemiology of America; Infectious Diseases Society of America. Clinical practice guidelines for Clostridium difficile infection in adults: 2010 update by the society for healthcare epidemiology of America (SHEA) and the infectious diseases society of America (IDSA). Infect Control Hosp Epidemiol. 2010;31(5):431-55.
17. Scottish Intercollegiate Guidelines Network (SIGN). Antibiotic prophylaxis in surgery. Edinburgh: SIGN; 2008. (SIGN publication no.104). [July 2008]. Available from URL: http://www.sign.ac.uk
18. NICE (2012) Infection: prevention and control of healthcare-associated infections in primary and community care Available at https://www.nice.org.uk/guidance/CG139 [NICE guideline].
19. Altman DG, Schulz KF, Moher D, Egger M, Davidoff F, Elbourne D, et al. The revised CONSORT statement for reporting randomized trials: explanation and elaboration. Annals Internal Medicine. 2001;134(8):663-94.
20. Liberati A, Altman DG, Tetzlaff J, Mulrow C, Gotzsche PC, Ioannidis JP, et al. The PRISMA statement for reporting systematic reviews and meta-analyses of studies that evaluate health care interventions: explanation and elaboration. Plos Med. 2009;6(7):e1000100.
21. Boutron I, Moher D, Altman DG, Schulz KF, Ravaud P. Extending the CONSORT statement to randomized trials of nonpharmacologic treatment: explanation and elaboration. Ann Intern Med. 2008;148(4):295-309.
22. Welch V, Petticrew M, Petkovic J, Moher D, Waters E, White H, Tugwell P; PRISMA-Equity Bellagio group. Extending the PRISMA statement to equity-focused systematic reviews (PRISMA-E 2012): explanation and elaboration. J Clin Epidemiol. 2015 Sep 5. pii: S0895-4356(15)00420-5.
23. Shamseer L, Moher D, Clarke M, Ghersi D, Liberati A, Petticrew M, Shekelle P, Stewart LA; PRISMA-P Group. Preferred reporting items for systematic review and meta-analysis protocols (PRISMA-P) 2015: elaboration and explanation. BMJ. 2015 Jan 2;349:g7647.
24. Chan AW, Tetzlaff JM, Gøtzsche PC, Altman DG, Mann H, Berlin JA, Dickersin K, Hróbjartsson A, Schulz KF, Parulekar WR, Krleza-Jeric K, Laupacis A, Moher D. SPIRIT 2013 explanation and elaboration: guidance for protocols of clinical trials. BMJ. 2013 Jan 8;346:e7586.
25. Moons KG, Altman DG, Reitsma JB, Ioannidis JP, Macaskill P, Steyerberg EW, Vickers AJ, Ransohoff DF, Collins GS. Transparent Reporting of a multivariable prediction model for Individual Prognosis or Diagnosis (TRIPOD): explanation and elaboration. Ann Intern Med. 2015 Jan 6;162(1):W1-73.
26. Martínez García L, Sanabria AJ, García Alvarez E, Trujillo-Martín MM, Etxeandia-Ikobaltzeta I, Kotzeva A, Rigau D, Louro-González A, Barajas-Nava L, Díaz Del Campo P, Estrada MD, Solà I, Gracia J, Salcedo-Fernandez F, Lawson J, Haynes RB, Alonso-Coello P; Updating Guidelines Working Group. The validity of recommendations from clinical guidelines: a survival analysis. CMAJ. 2014;186(16):1211-9.
27. Treweek S, Oxman AD, Alderson P, Bossuyt PM, Brandt L, Brożek J, Davoli M, Flottorp S, Harbour R, Hill S, Liberati A, Liira H, Schünemann HJ, Rosenbaum S, Thornton J, Vandvik PO, Alonso-Coello P; DECIDE Consortium. Developing and Evaluating Communication Strategies to Support Informed Decisions and Practice Based on Evidence (DECIDE): protocol and preliminary results. Implement Sci. 2013;8:6.
28. Kristiansen A, Brandt L, Alonso-Coello P, et al. Development of a novel electronic multilayered presentation format for clinical practice guidelines. CHEST 2014, early online September 2014
29. Schünemann HJ, Al-Ansary LA, Forland F, Kersten S, Komulainen J, Kopp IB, Macbeth F, Phillips SM, Robbins C, van der Wees P, Qaseem A; Board of Trustees of the Guidelines International Network. Guidelines International Network: Principles for Disclosure of Interests and Management of Conflicts in Guidelines. Ann Intern Med. 2015;163(7):548-53.
30. Grupo de trabajo sobre actualización de GPC. Elaboración de Guías de Práctica Clínica en el Sistema Nacional de Salud. Actualización del Manual Metodológico. March 2016. Available: http://portal.guiasalud.es/emanuales/elaboracion_2/
31. Hasenfeld R, Shekelle PG. Is the methodological quality of guidelines declining in the US? Comparison of the quality of US Agency for Health Care Policy and Research (AHCPR) guidelines with those published subsequently. Qual Saf Health Care. 2003;12(6):428-34.
32. Moher D, Schulz KF, Simera I, Altman DG. Guidance for developers of health research reporting guidelines. Plos Med. 2010;7(2):e1000217.
33. Moher D, Weeks L, Ocampo M, Seely D, Sampson M, Altman DG, Schulz KF, Miller D, Simera I, Grimshaw J, Hoey J. Describing reporting guidelines for health research: a systematic review. J Clin Epidemiol. 2011;64(7):718-74
